# Incidental Finding of Isolated Accessory Mitral Valve Tissue in Two Adults

**DOI:** 10.1002/ccr3.9699

**Published:** 2024-12-06

**Authors:** Zhaofen Wang, Fang Du, Hao Zhu, Yuzhe Song, Lijuan Huang, Yi Yan, Peng Chang

**Affiliations:** ^1^ The Second Hospital and Clinical Medical School Lanzhou University Lanzhou China; ^2^ Department of Cardiology Lanzhou University Second Hospital Lanzhou China; ^3^ Department of Radiology Lanzhou University Second Hospital Lanzhou China

**Keywords:** accessory mitral valve tissue, cardiac computed tomography, case report, echocardiography

## Abstract

Accessory mitral valve tissue is a rare congenital anomaly often linked to the anterior mitral leaflet, diagnosed via echocardiography. It may cause left ventricular outflow tract obstruction, with prognosis depending on obstruction severity and regular monitoring essential in asymptomatic cases.

## 1 Introduction

Accessory mitral valve tissue (AMVT) is an uncommon congenital abnormality in the structure of the heart, usually found in childhood and rarely detected in adulthood. Frequently, it is found alongside other inborn abnormalities of the heart and blood vessels, such as flaws in the walls between the ventricles and atria, narrowing of the aorta below the valve, irregularities in the coronary arteries, and constriction of the aorta. AMVT is often discovered coincidentally, with certain patients continuing without symptoms, exhibiting solely a cardiac murmur. However, AMVT can lead to the obstruction of the left ventricular outflow tract obstruction (LVOTO), and the prognosis depends on the severity of the obstruction. The symptoms might vary and include dyspnea, chest discomfort, palpitations, tiredness, syncope, and embolism. Echocardiography, namely, three‐dimensional and transesophageal echocardiography (TEE), plays a vital role in the diagnosis of AMVT. It is often connected to the anterior mitral leaflet (AML), as indicated in multiple studies. This report focuses on two examples with AMVT: one involving the anterior mitral valve and the other involving the posterior mitral valve. These cases were identified during assessments for palpitations and chest discomfort after physical activity, respectively. Neither patient exhibited LVOTO or any other cardiac irregularities, resulting in the decision to conduct regular monitoring.

## 2 Case Report

### 2.1 Case 1

A 50‐year‐old man experiencing month‐long palpitations was admitted to our hospital. Physical examination revealed irregular rhythm and a mitral systolic murmur of 2+/6+. Resting electrocardiography showed ventricular ectopic beats.

**FIGURE 1:**
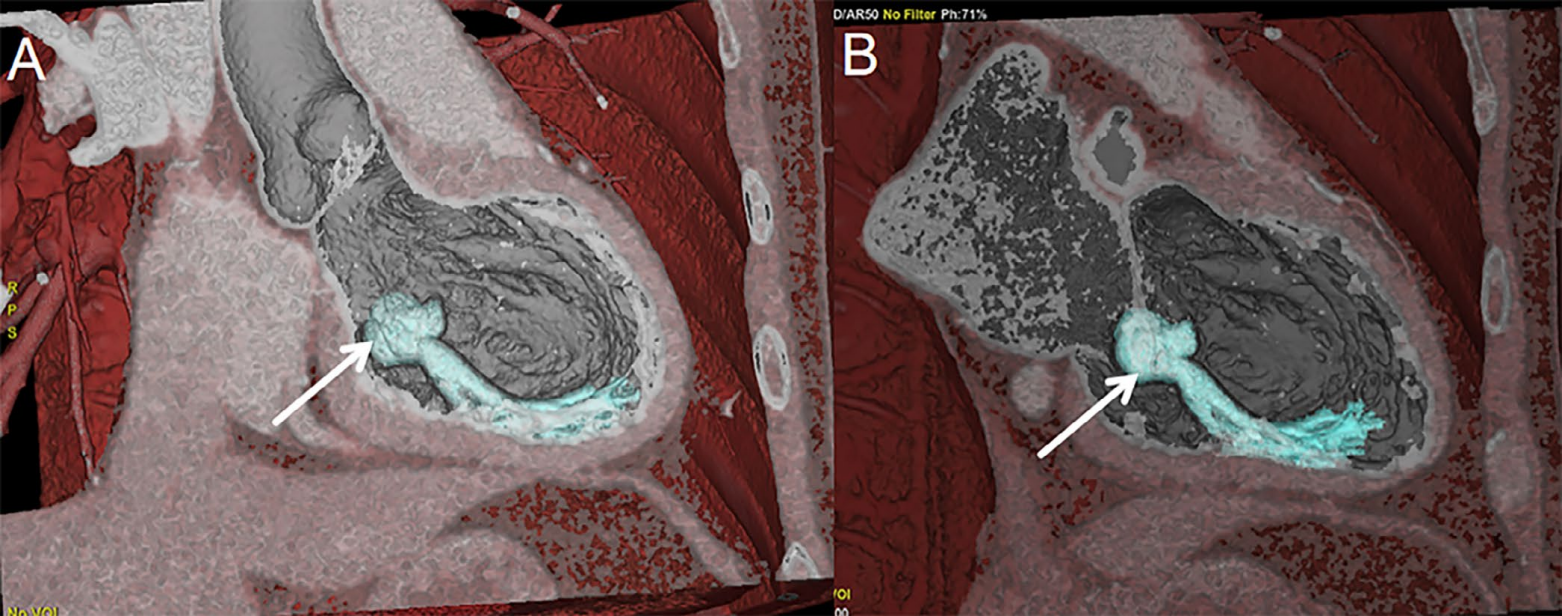
The features of case 1 on cardiac CT. Through cardiac CT (A, B), it can be seen that circular tissue resembling myxomas and fibroids attached to the mitral valve, as well as tendon‐like structures connected to this tissue, is attached to the ventricular wall (shown by the white arrow in the figure).

**FIGURE 2:**
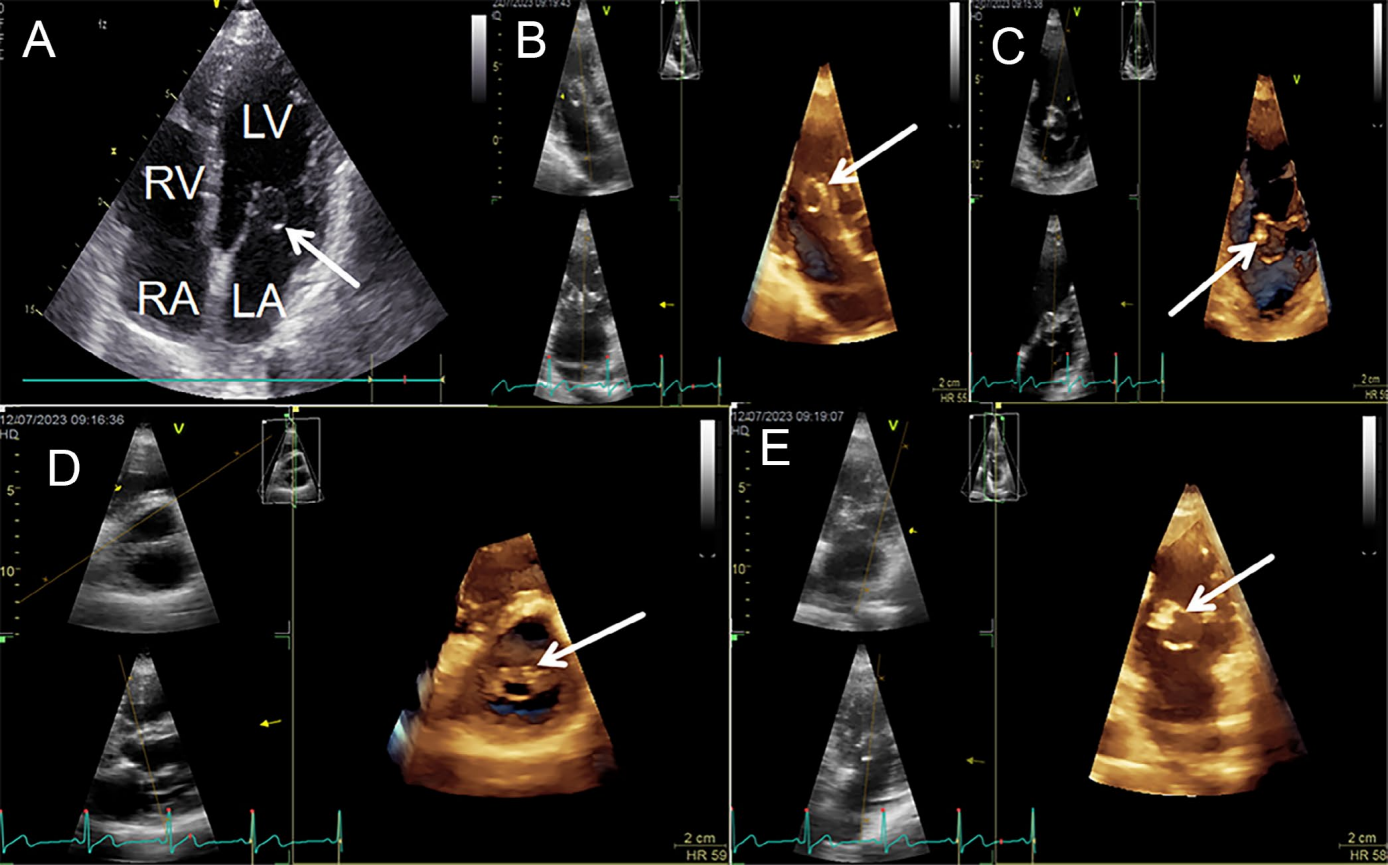
The features of case 1 on 2D and 3D TTE. On 2D (A) and 3D TTE (B–E) reveals a 15 × 12 mm mass located over the anterior mitral valve with a normal valve area of 2.7cm^2^ and appears a large, round, and soft mass with smooth borders and situated in the anterior leaflet of the mitral valve (shown by the white arrow in the figure). LA, left atrium; LV, left ventricle; RA, right atrium; RV, right ventricle.

Cardiac computed tomography (cardiac CT) scans did not reveal any significant abnormalities in the coronary arteries or the mitral valve orifice. However, a suspicious mass at the mitral valve orifice, potentially indicative of a mucinous tumor or leiomyosarcoma, remained undiagnosed (Figure 1). Subsequent transthoracic echocardiography (TTE) identified a 15 × 12 mm mass on the anterior mitral valve leaflet, with a normal valve area of 2.7cm^2^. Both 2D and 3D TTE showed a large, round, and soft mass with smooth contours on the mitral valve's anterior leaflet. The mobile mass featured central echolucent areas resembling liquefaction without acoustic shadowing (Figure 2), and was initially thought to be a cystic lesion of the anterior mitral valve leaflet, possibly a hematogenous cyst.

**FIGURE 3:**
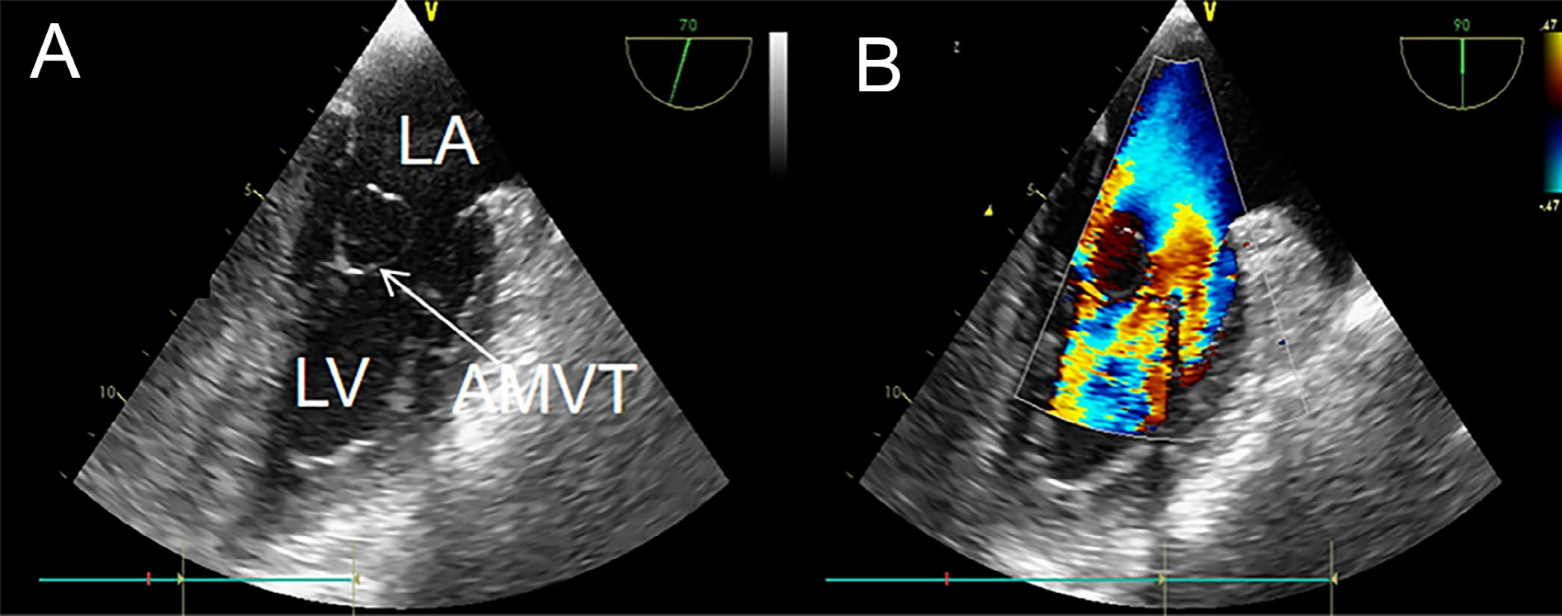
The features of case 1 on TEE and color Doppler. Systolic (A) phases of TEE can better display the anterior structure (shown by the white arrow in the figure). Color Doppler (B) shows no blood flow in the center of the mass and mild mitral regurgitation. LA, left atrium; LV, left ventricle.

Transthoracic echocardiography provided enhanced visualization of anterior structures (Figure 3A). The mass appeared with a central non‐echo‐dense area akin to liquefaction, surrounded by a hyperechoic border, without any flow in the central zone as per color Doppler analysis. It was attached to the A2–A3 scallops of the anterior mitral valve leaflets without impeding valve motion and flow. Mild mitral regurgitation was observed, indicated by a vena contracta width of 2.3 mm (Figure 3B).

Based on the morphology and characteristics observed in echocardiography, the patient was diagnosed with AMVT. Given the absence of other cardiovascular abnormalities, pathology, or significant left ventricular outflow tract obstruction, a conservative approach of dynamic observation and regular follow‐up was recommended.

### 2.2 Case 2

A 71‐year‐old man came to our hospital complaining of chest discomfort after physical exertion. Physical examination revealed systolic murmur radiating from the aortic region to the suprasternal region, decreased S2 tone, normal pulses, and symmetrical blood pressure.

**FIGURE 4:**
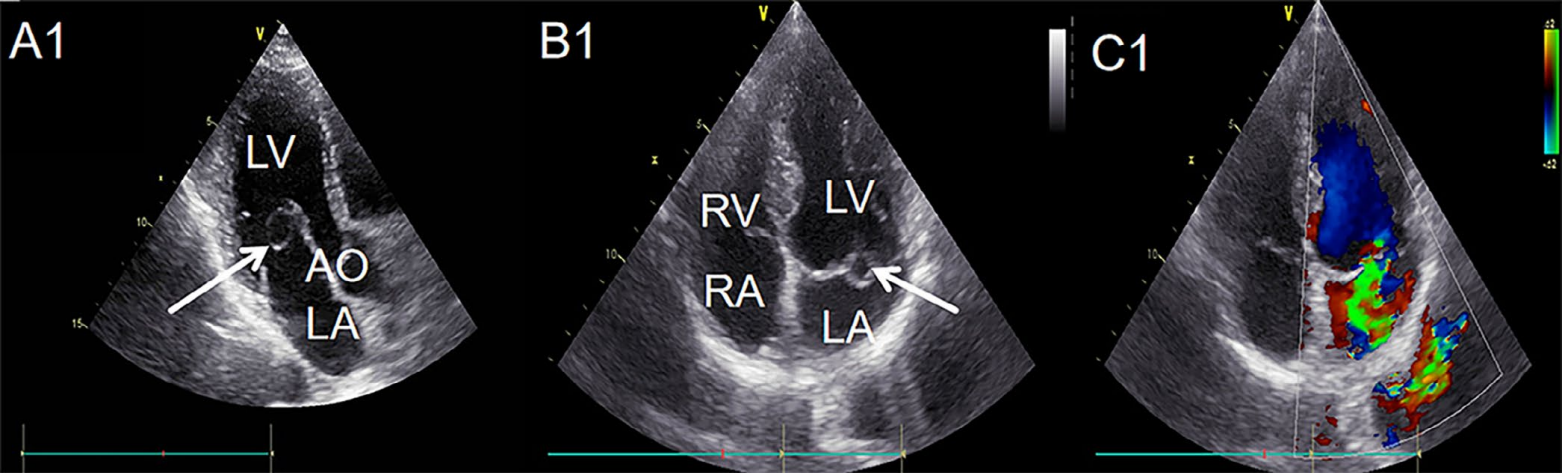
The features of case 2 on TTE and color Doppler. From the figure (A1, B1), it can be clearly seen that there is a small, round mass above the posterior part of the mitral valve, with a smooth boundary (shown by the white arrow in the figure). Color Doppler (C1) shows mitral regurgitation. LA, left atrium; LV, left ventricle; RA, right atrium; RV, right ventricle.

The echocardiographic evaluation showed that the internal diameters of each atrioventricular cavity were within normal limits. Additionally, the ventricular wall segments had normal thickness without evidence of left ventricular hypertrophy, and normal motion and contraction amplitudes were observed. TTE identified a 9.5 × 6 mm mass on the posterior mitral valve. This mass was small, round, and soft, with smooth edges and central echolucent areas, lacking any acoustic shadowing artifacts (Figure 4A1,B1). A tendon‐like structure with pronounced echogenicity was also noted within the left ventricular cavity.

Based on its echocardiographic features, the patient was diagnosed with AMVT. The remaining valves appeared normal in morphology and structure. The aortic root diameter was within normal limits, and the pericardial cavity appeared normal. Color Doppler imaging (Figure 4C1) revealed moderate mitral regurgitation alongside mild aortic regurgitation. The mitral E/A ratio was recorded at 0.65/0.86 m/s, indicating a reduced left ventricular diastolic function with an E/A ratio less than 1. However, the patient showed no signs of left ventricular outflow tract obstruction or other congenital cardiac anomalies. Consequently, a decision was made to continue monitoring the patient's condition. There were no symptoms after 2 years of follow‐up.

## 3 Methods

We searched the PubMed databases for the key terms “Accessory mitral valve tissue” and “Accessory mitral valve” from May 1972 [1] to September 2023 [2]. References of the included studies were also searched for potentially relevant articles. Studies of AMVT were collected and analyzed, respectively, before and after 2012 [3], including demographic characteristics and concomitant structural anomalies. Different imageological examination features for AMVT are summarized according to relevant studies.

In our case reports, all echocardiographic examinations were performed by qualified cardiologists using the same equipment (Vivid E9, GE Healthcare, Waukesha, WI) and transducers (4 V [1.7/3.3 MHz], [M5S 1.7/3.4 MHz] and 6Tc [6.0/6.0 MHz]).

The patient examinations were all performed using a 256‐row widescope CT scanner (Revolution CT, GE Healthcare). Coronary computed tomography angiography (CCTA) acquisition included all levels from 1 cm below the tracheal bifurcation to the bottom of the heart and was triggered via smart tracking, with the region of interest placed in the ascending aorta. The contrast agent iopromide (370 mg/mL) was injected 0.9 mL/kg into the median cubital vein at a rate of 5.0–5.5 mL/s, followed by a 40 mL normal saline rinse at the same rate. CCTA was acquired with prospective electrocardiographic gating and set up as follows: tube voltage = 100 kvp, tube current = 400–700 mA, scanning field of view = 36 cm, display field of view = 24 cm, matrix = 512 × 512, rotation time = 0.28 S, and slice thickness = 0.625 mm. The reconstruction parameters were smooth kernel (STANDARD) and 60% of adaptive statistical iterative reconstruction Veo.Advanced Workstation 4.7 (AW4.7; GE Healthcare).

## 4 Discussion

Accessory mitral valve tissue refers to the existence of extra sections and fragments of valvular structures that are attached to the normal mitral valve apparatus (including the AML, posterior mitral leaflet, tendon cords, anterior lateral papillary muscles, and posterior medial papillary muscles) in the chambers of the left side of the heart [4]. The concept of AMVT was initially defined by Chevers in 1842 and the first recorded surgical instance of AMVT occurred in 1963 [5]. Research suggests that approximately 1 in every 26,000 adults might be affected by this condition [6]. The true prevalence of AMVT could be higher than reported, as its rarity often leads to misdiagnosis or delayed identification. AMVT exhibits a variety of shapes, commonly described in the literature as cystic, balloon‐like, parachute‐like, sail‐like, lobular, sheet‐like, membranous, or pedunculated [4]. The specific shape of AMVT largely depends on its attachment site within the left heart structures.

Prifti et al. [7] divided AMVT into two categories based on its morphology: fixed type I and mobile type 2. Type II encompasses pedunculated (type IIA) and membranous (type IIB) varieties. In our patient's case, AMVT was identified as mobile with a balloon‐like appearance, closely aligning with the characteristics of type IIB, which is the most common form of AMVT, according to Prifti et al.'s morphological classification. Yetkin et al. [4] proposed a different categorization, using the mitral valve leaflets as anatomical reference points. Their classification consists of three groups: Type I AMVT attaches above the level of the leaflets; type II is found on the mitral valve leaflets; and type III attaches below the leaflets.

The exact cause of AMVT remains unclear, but it is believed to stem from incomplete separation of the mitral valve from the endocardial cushion or residual endocardial cushion material not fully merged with the mitral leaflets [8, 9]. The presence of congenital cardiac and vascular anomalies, such as septal defects, subaortic valves and stenosis, and atrial septal aneurysms, supports this theory [5, 10]. Histological examination of excised AMVT tissue typically reveals a relatively normal valve structure comprising endothelial, fibrous, and spongy layers [10]. However, deviations from this norm, such as mucinous degeneration and infiltration of inflammatory cells, have been observed [11].

The manifestation of symptoms in patients with AMVT is largely influenced by several factors: accompanying congenital cardiovascular anomalies, the extent of LVOT, any associated complications, and the specific location where AMVT is attached [12]. Commonly, it is believed that symptoms in AMVT patients become prominent when the mean LVOT pressure gradient exceeds 50 mmHg. However, our review of the data reveals a different perspective. Among patients with a documented LVOT pressure gradient, symptoms were exhibited despite having an average pressure gradient below the 50 mmHg threshold. This indicates that symptoms in patients with AMVT can manifest even at lower levels of LVOT obstruction. In those who are symptomatic, the nature and severity of their clinical presentation primarily depend on the degree of LVOT obstruction. Embolism, a rare but severe complication of AMVT, can occur, regardless of the presence of LVOTO, and includes a range of manifestations like cerebrovascular embolism [13], retinal artery embolism [14, 15], transient ischemic attack [6], stroke, and systemic embolism, leading to conditions such as right hemiparesis [16]. This finding underscores the variability in how AMVT can present clinically and the importance of considering individual patient characteristics in its management.

Echocardiography is crucial in diagnosing AMVT, as it is particularly essential for imaging purposes. The diagnosis of AMVT primarily relies on distinct echocardiographic features [2] such as the visual identification of the mass extending through and retracting from the aortic valve throughout the systolic and diastolic phases of the heart. Another vital factor involves determining where the mass is connected to the mitral valve, such as AML or PML. Furthermore, the unique visual characteristics of the mass, such as its cystic or pedunculated nature, contribute to the precise recognition of AMVT. These combined echocardiographic characteristics allow clinicians to diagnose and evaluate the illness accurately.

Vegetations, cardiac tumors (such as mucinous tumors), and mitral valve aneurysms have similar echocardiography features to AMVT. Still, they originate in the myocardium or accumulate on the low‐pressure side of the cardiac valves and do not have an AMVT appearance [7] The subaortic membrane is the same but fissure‐like or membranous and creates an LVOT gradient [17]. Clinicians must also be aware that asymptomatic individuals without LVOTO at rest may undergo exercise stress echocardiography to rule out dynamic gradients [11, 18] Because LVOTO might be latent, only exercise or heart‐exciting medications like dobutamine can activate it [11].

Laguna et al. [19] documented a 48‐year‐old male patient, who had a subaortic resection and had exertional syncope before being diagnosed with AMVT. AMVT was misdiagnosed as an AML tendon cord rupture, according to Panduranga et al. [20] Patients with suspected mitral tendon cable rupture without regurgitation should seek AMVT. Similar to instance 1, TTE initially misdiagnosed it as a haematocyst, but TEE later identified it as an AMVT. In addition to focusing on the patient's clinical presentation (especially for patients with a cardiac murmur who are otherwise healthy and in patients with LVOT obstruction or tissue leading to subaortic stenosis and unexplained embolism), the differential diagnosis of this disease is rare. Many situations require close attention to avoid AMVT misdiagnosis and delay.

According to the literature, the treatment of AMVT consists of conservative and surgical therapies in general. However, since AMVT symptoms range from asymptomatic to life‐threatening, it is not always easy to determine the appropriate time for surgical intervention.

Follow‐up and prophylactic surgery for individuals without symptoms, LVOTO, and other cardiovascular anomalies or pathology have varied viewpoints. Prophylactic surgery can prevent AMVT problems, but persistent LVOTO or associated congenital malformations result in an 8.9% death rate following surgery [18]. Surgery is necessary for individuals with symptomatic LVOT blockage, other congenital cardiac defects, valve disease, and heart failure, even though the best adaption period is debated. Resection of the AMVT and detachment of the accessory tendon cords is the preferred treatment [5]; however, the mitral valve function must be evaluated first. AMVT complexity determines surgical access. It usually includes transaortic [21, 22], left atriotomy, [19] or both [11, 23, 24]. Besides surgery, β‐blockers [25] can also be effective for patients experiencing symptoms after exercise.

This report concludes that this unusual instance of AMVT, whether it has had surgery or not, lacks follow‐up data and duration. Thus, doctors should improve AMVT patient follow‐up in future clinical work to improve prognosis.

## 5 Conclusions

Accessory mitral valve tissue is increasingly diagnosed in adults, though it is more common in children, and isolated cases are on the rise. Diagnosis relies heavily on echocardiography, particularly TEE, to distinguish AMVT from other congenital heart anomalies. Due to limited data, the long‐term prognosis of asymptomatic patients without surgery remains uncertain, highlighting the importance of continuous follow‐up.

## Author Contributions

**Peng Chang:** funding acquisition, investigation, project administration, resources, visualization. **Zhaofen Wang:** writing – original draft. **Fang Du:** methodology, resources, software. **Hao Zhu:** methodology, resources, software. **Yuzhe Song:** data curation. **Lijuan Huang:** data curation. **Yi Yan:** data curation.

## Ethics Statement

This paper includes two patients with AMVT from the Department of Cardiovascular Medicine at the Second Hospital of Lanzhou University. Both patients provided written informed consent with the approval of their families, after which their clinical data and medical imaging were collected. The study protocol was reviewed and approved by the Medical Ethics Review Committee of the Second Hospital of Lanzhou University (Project No. 2024A‐266). All procedures involving human participants adhered strictly to the ethical standards set by institutional and/or national research committees.

## Conflicts of Interest

The authors declare no conflicts of interest.

## Data Availability

All data will be made available upon reasonable request to the corresponding author.
